# Molecular Characterization of Tickborne Relapsing Fever *Borrelia,* Israel

**DOI:** 10.3201/eid1211.060715

**Published:** 2006-11

**Authors:** Marc Victor Assous, Amos Wilamowski, Herve Bercovier, Esther Marva

**Affiliations:** * Ministry of Health, Jerusalem, Israel;; † Hebrew University–Hadassah Faculty of Medicine, Jerusalem, Israel

**Keywords:** Borrelia, Borrelia persica, relapsing fever, TBRF, tick, Ornithodoros tholozani, taxonomy, flaB, flagellin, molecular characterization, Israel, cave, Middle East, dispatch

## Abstract

Blood samples from 18 tickborne relapsing fever (TBRF) patients and *Ornithodoros tholozani* specimens were tested with a *Borrelia fla*B-PCR. Results were positive for all patients and 2%–40% of ticks. A 7–amino acid gap characterized all 9 sequenced flagellin gene amplicons. By phylogenetic analysis, Israel TBRF *Borrelia* sequences clustered separately from American and African groups.

Tickborne relapsing fever (TBRF) is caused by Borrelia species and is transmitted to humans by Ornithodoros soft ticks. Worldwide, a dozen Borrelia species are known to cause this disease (*1*). In Israel, TBRF is considered to be caused by Borrelia persica and transmitted by the cave tick Ornithodoros tholozani (*1*). This tick and TBRF are distributed through Central Asia (*2*) and the Middle East (*1*). Other Borrelia species that cause TBRF have been described in Iran (*3*), but their precise range of distribution is not known.

In Israel, from 1980 through 2002, 184 cases of TBRF were reported among the civilian population (8 cases/year), and 88% of the case-patients were infected in caves (*4*). Among military personnel, TBRF incidence averages 6.4 cases/100,000 persons (*5*). In Jordan, an average of 72 civilian cases per year was reported from 1959 through 1969 (*6*). In Iran, an average of 100 cases per year has been recently reported (*7*).

TBRF in Israel was first reported by Nicholson (*8*) at the time World War I. Detailed clinical and epidemiologic features of the disease are well described in this article, particularly the transmission by ticks (*8*). However, Nicholson incorrectly attributed the disease to the soft tick Argas persicus. In 1937, Adler et al. clearly identified O. papillipes (tholozani) as the vector of the disease (*9*) and characterized the causative agent as Spirochaeta persica (*10*).

Although American (*11*) and African TBRF Borrelia (*12*) are now better characterized, no definitive molecular characterization of Borrelia species responsible for relapsing fever has been achieved in Israel. The aim of this study is to provide initial molecular characterization of the etiologic agent of TBRF in Israel from both ticks and human samples and to compare it with previously described agents of TBRF in other parts of the world.

## The Study

**Figure 1:**
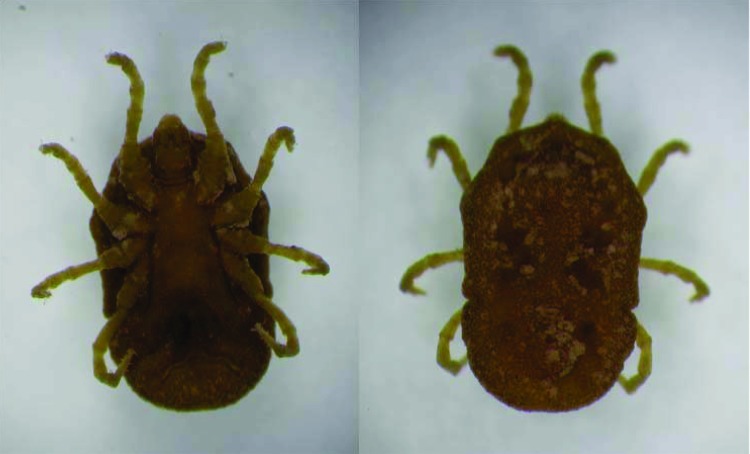
*Ornithodoros tholozani* ventral (A) and dorsal (B) views.

We designed a genus-specific set of primers (BOR1: 5´ TAA TAC GTC AGC CAT AAA TGC 3´ and BOR2: 5´ GCT CTT TGA TCA GTT ATC ATT C 3´) that targeted the Borrelia flaB flagellin gene (*13*). Each PCR mixture (25 μL) contained 3 μL of target DNA and was subjected to 1 min at 95°C, followed by 40 cycles of 56°C for 30 sec, 72°C for 30 sec, 94°C for 30 sec, and 5 min at 72°C for final elongation. DNA of B. duttonii and B. burgdorferi sensu stricto (strain B31) was used as controls. DNA of blood and ticks was extracted with the DNA easy tissue kit (Qiagen, Hilden, Germany). Tick samples were collected by using CO_2_ traps in caves and were identified as Ornithodoros tholozani (Figure 1) by the Entomology Laboratory (Ministry of Health, Jerusalem). The tick specimens collected were tested either individually or as pools. Of 184 tick specimens collected from 5 different areas (Table 1), 94 were tested by BOR1-BOR2 PCR. One pool of 5, a pool of 4, and 6 individual specimens were positive; all produced a unique band 750 bp in length. The percentage of tick infection was variable, ranging from <2% in Ma'ale-Adumim to 40% in the Be'er Sheva region.

**Table 1:** Percentage of tick infestation by PCR at several locations

Region/location	No. ticks collected	No. tested	Pools/individuals	PCR result	% Infestation
Jerusalem outskirts					
Ma'ale Adumim	51	51	9 individuals	–	<1.9
			5 pools of 5	–	
			3 pools of 4	–	
			5 individuals	–	
Jericho					
Makoh	30	10	Pool of 4	–	6.6
			6 individuals	1+	
Center					
Gimzo	45	15	Pool of 5	+	13–40
			Pool of 3	–	
			Pool of 3	–	
			4 individuals	1+	
Tiberias					
Migdal	8	8	Pool of 4	+	12.5–50
			Pool of 4	–	
Be'er Sheva, Arad Valley					
Hurvat Kasif	50	10	10 individuals	4 positive	40

For patients, the TBRF diagnosis was established as previously reported (*5*). Eighteen samples of human blood were sent to the Parasitology Reference Center (Ministry of Health, Jerusalem); the samples corresponded to 15 confirmed cases (positive blood smear) and 3 associated cases of TBRF (negative blood smear). On receipt at the laboratory, fresh human blood samples were examined by darkfield microscopy for viable Borrelia and, if detected, 200 μL of blood was injected into 10-mL vials of BSK-H medium (*14*) and into 10-week-old ICR mice by the intraperitoneal route. In 4 patients, blood examined by darkfield microcopy showed 1–5 motile Borrelia per slide. In vitro cultivation was unsuccessful. However, Borrelia (1–5/field) were detected on day 4 (twice) and day 6 (twice) in the blood of mice injected intraperitoneally with patient blood. Cultivation attempts from positive mice blood were also unsuccessful. In contrast, all the samples were found positive by BOR1-BOR2 PCR, showing a unique band of 750 bp (data not shown).

PCR products were cloned in T7 plasmid by pGEM-T Easy vector SystemII (Promega, Madison, WI, USA). Plasmids containing inserts were purified and sent for 2-strand sequencing. Direct sequencing of DNA amplified by the BOR1 and BOR2 primers was performed later.

Phylogenetic and molecular evolutionary analyses were conducted by using MEGA version 3.1 (*15*). Among published flaB genes of TBRF Borrelia strains, only sequences for which a translated protein existed were taken in account. Because of the large number of available sequences for American Borrelia associated with TBRF, as well as for B. duttonii and B. recurrentis, a single sequence representative of each cluster was chosen for taxonomic analyses.

Three PCR amplicons (from 1 tick and 2 human samples) were sequenced after cloning, whereas 6 amplicons (from 2 ticks and 4 human samples) were analyzed by direct sequencing. These 9 sequenced samples showed 98%–100% homology between them and could be divided into 3 groups. The same DNA sequence was found in tick TG52 and in blood from 2 patients, HumanBlood2 and HumanBlood4. These 3 sequences had an additional triplet at the position 627. The second group of sequences, which consisted of tick samples TGd1 and CBkc7 and blood samples C1025B, FL1, and HumanBlood3, were identical, with only 3 minor substitutions between them. The third group consisted of the HumanBlood1 sample.

**Figure A1:**
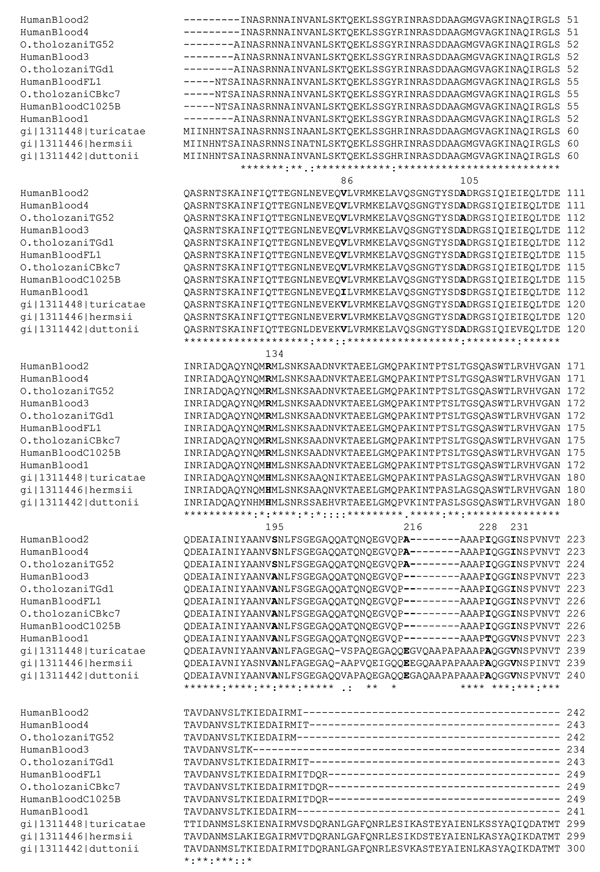
CLUSTAL W multiple alignment of 9 Israeli tickborne relapsing fever sequences (bold letters indicate variable amino acid positions as shown in Table 2). The GenBank accession numbers for nucleotide sequences of Borrelia persica flaB shown here are as follows: HumanBloodFL1 (DQ673617), HumanBloodC1015B (DQ679904), OtholozaniCBkc7 (DQ679905), HumanBlood1 (DQ679906), HumanBlood2 (DQ679907), HumanBlood3 (DQ679908), HumanBlood4 (DQ679909), OtholozaniTG52 (DQ679910) and OtholozaniTGd1 (DQ679911).

All the translated sequenced amplicons showed a very specific signature by the presence of a 7–amino acid (aa) gap at position 216 (Figure A1) when compared with previously described TBRF Borrelia flaB genes. In addition, the local TBRF Borrelia sequences could be grouped into 3 subtypes, according to variation at 7-aa positions (Table 2).

**Table 2:** Variable amino acid (aa) positions and type definition of the *flaB* gene for the 9 samples sequenced for the Israeli tickborne relapsing fever *Borrelia persica*

Type	Position*	No. strains


86	105	134	195	216	228	231
I	V	A	R	A	-	I	I	5
II	V	A	R	S	A	I	I	3
III	I	S	H	A	-	T	V	1

*Numbering according the aa sequence of *B. hermsii* gi|1311448|.

**Figure 2:**
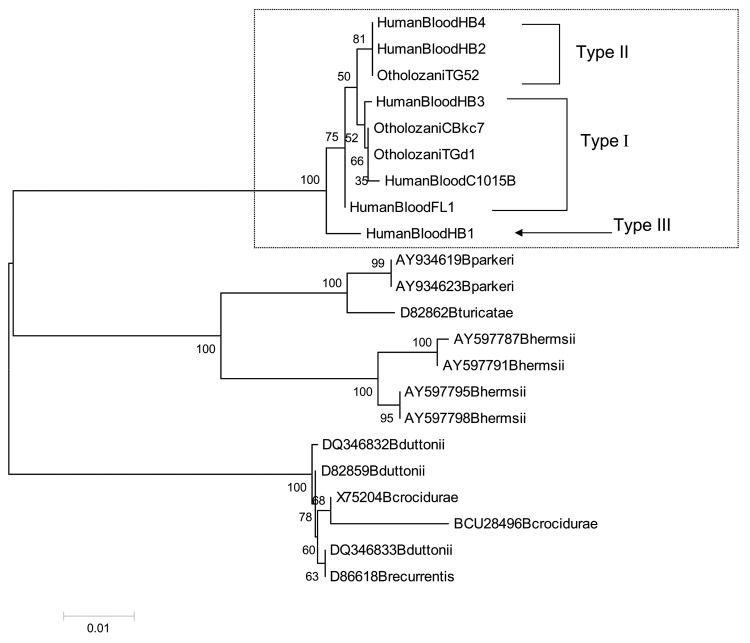
Phylogenetic tree based on *fla*B nucleotide sequences. The tree was constructed by the neighbor-joining method in a pairwise deletion procedure. Distances were calculated according to the Jukes and Cantor method. Numbers at nodes correspond to the percentage confidence level in a bootstrap test performed on 1,000 replicates. The scale bar corresponds to a 0.01 distance. The GenBank accession numbers for nucleotide sequences of *Borrelia persica fla*B shown here are as follows: HumanBloodFL1 (DQ673617), HumanBloodC1015B (DQ679904), OtholozaniCBkc7 (DQ679905), HumanBlood1 (DQ679906), HumanBlood2 (DQ679907), HumanBlood3 (DQ679908), HumanBlood4 (DQ679909), OtholozaniTG52 (DQ679910), and OtholozaniTGd1 (DQ679911).

Comparison with published flaB protein sequences of TBRF Borrelia showed 88%–90% homology with B. duttonii and B. recurrentis, 85%–90% with B. crocidurae, 86%–88% with B. turicatae, 87%–89% with B. hermsii, and 85%–88% with B. parkeri. The sequences of the Israel TBRF Borrelia isolated from different samples clearly clustered in a separate group from the American and the African TBRF species (Figure 2).

## Conclusions

Our results suggest that infection rates differ according to location, despite the small number of ticks tested and the use of pools. BOR1-BOR2 PCR was more sensitive than blood smear examination (100% vs 83%). An identical DNA sequence was found in both tick and patient samples and thus confirms, at the molecular level, the role of O. tholozani as the vector of TBRF in Israel.

A signature (7-aa gap) of the flaB flagellin defined the Israeli TBRF sequences as a homologous group different from other TBRF species. Despite the small number of samples studied, a clear polymorphism existed also at the protein level, resulting in 3 local types. This diversity can be explained by the use of direct sequencing of samples rather than through cultivation that reduces the biodiversity of isolates by selecting the most successful in vitro clone.

This study opens a new avenue in TBRF Borrelia studies by demonstrating a Middle East cluster in addition to the American and African groups. These results open encouraging perspectives for the better understanding of entomologic, epidemiologic, and bacteriologic aspects of this disease and may contribute to better diagnosis and treatment.
